# Rapid visualization of *Clostridioides difficile* toxins A and B by multiplex RPA combined with CRISPR-Cas12a

**DOI:** 10.3389/fmicb.2023.1119395

**Published:** 2023-03-08

**Authors:** Tong Jiang, Xinyi Hu, Chunhui Lin, Zhaoxin Xia, Wensu Yang, Yi Zhu, Huaming Xu, Hao Tang, Jilu Shen

**Affiliations:** ^1^The First Affiliated Hospital of Anhui Medical University, Hefei, Anhui, China; ^2^Anhui Public Health Clinical Center, Hefei, Anhui, China

**Keywords:** *Clostridioides difficile*, toxin gene, recombinase polymerase amplification, CRISPR/Cas12a, lateral flow strip, point-of-care testing

## Abstract

**Purpose:**

*Clostridioides difficile* (*C. difficile*) infection is the most common cause of nosocomial infection, which is a severe challenge in modern medical care. Currently, many laboratory diagnostic methods for *C. difficile* are available, such as PCR, culture-based tests, and antigen-based tests. However, these methods are not suitable for rapid point-of-care testing (POCT). Therefore, it is of great significance to develop a rapid, sensitive, and cost-effective method to detect *C. difficile* toxin genes.

**Methods:**

Recently, the development of clustered regularly interspaced short palindromic repeats (CRISPR) technology has emerged as a promising tool for rapid POCT. In this study, we developed a rapid and specific detection platform for dual *C. difficile* toxins by combining recombinase polymerase amplification (RPA) and CRISPR/Cas12a.

**Results:**

The platform includes multiplex RPA-cas12a-fluorescence assay and multiplex RPA-cas12a-LFS (Lateral flow strip) assay, through which the detection limit for tcdA and tcdB was 10 copies/μL and 1 copy/μL, respectively. The results can be more clearly distinguished using a violet flashlight, which realized a portable visual readout. The platform can be tested within 50 min. Furthermore, our method did not cross-react with other pathogens that cause intestinal diarrhea. The results of 10 clinical samples using our method was 100% consistent with those from real-time PCR detection.

**Conclusion:**

In conclusion, the CRISPR-based double toxin gene detection platform for *C. difficile* is an effective, specific, and sensitive detection method, which can be used as a powerful on-site detection tool for POCT in the future.

## Introduction

*Clostridioides difficile* (*C. difficile*) is the main pathogen of nosocomial infection (Khanna et al., 2012). First isolated from infant feces by Hall and O’Toole (1935), *C. difficile* was found to be a conditional pathogenic bacterium that exists in human and animal intestines. The abuse of antibiotics causes imbalanced intestinal flora, leading to the overgrowth of *C. difficile* that secrets extensive toxins, which further causes the onset of *C. difficile* infection (CDI), including antibiotic-associated diarrhea (AAD), pseudomembranous colitis (PMC), toxic megacolon, and sepsis etc. (Rodriguez-Pardo et al., 2013; Sun and Hirota, 2015; Avila et al., 2016; Kuy et al., 2016; Unal and Steinert, 2016). It has been reported that 25 to 33% of antibiotic-associated diarrhea and almost 100% of pseudomembranous colitis are caused by *C. difficile* infection (Butala and Divino, 2010; Smits et al., 2016; Bartlett, 2017). The causative toxin factors associated with *C. difficile* are mainly toxin A (TcdA) and toxin B (TcdB) (Chumbler et al., 2016; Di Bella et al., 2016), which could be simultaneously secreted by a large proportion of clinically isolated strains. While some strains have been reported to be TcdA-negative and TcdB-positive (van den Berg et al., 2004), some strains can also produce additional binary toxins (*C. difficile* transferase, CDT) (Bauer et al., 2011). With the recent outbreak of high-yielding strains (BI/NAP1/027 type) in Europe and North America, the morbidity and mortality of CDI and drug resistance have increased significantly (O’Connor et al., 2009). As *C. difficile* infection becomes a worldwide public health problem, the accurate diagnosis of *C. difficile* infection is crucial for both infection control and prognosis.

At present, there are many laboratory diagnostic methods for *C. difficile*, such as anaerobic culture method, glutamate dehydrogenase (GDH) assay, toxin A/B enzyme immunoassay (EIA), cell cytotoxicity neutralization assay (CCNA), and nucleic acid amplification test (NAAT) etc. (Chen et al., 2017). CCNA is currently the gold standard for diagnosis of CDI, but it is not suitable for rapid clinical detection due to the long testing period and the requirement for professional knowledge and strict operation (Chen et al., 2017; Nagy, 2018).

Antigen detection has become a widely used method due to its low cost and simple operation, but the sensitivity of this method requires further improvement. NAAT has been greatly improved in recent years and has become a popular diagnostic method. Compared with CCNA, the sensitivity of NAAT is remarkably higher, while the detection time is significantly reduced. The automated devices such as GeneXpert are also available for NAAT. However, NAAT requires expensive equipment and complex thermal cyclers, which obviously hinders its application in point-of-care or resource-limited areas. Therefore, there is an urgent need to develop a rapid, highly specific, and highly sensitive method for detecting *C. difficile* that does not require special equipment.

Recently, clustered regularly interspaced short palindromic repeats (CRISPR)/Cas (CRISPR-associated) proteins, namely CRISPR-Cas systems, have shown great promise in gene editing and molecular diagnosis due to their high sensitivity, specificity, and ease of operation (Kaminski et al., 2021; Liu et al., 2022). By recognizing a unique TTTN-containing protospacer-adjacent motif (PAM) sequence, some Cas nucleases such as Cas12a have been shown to cleave double-stranded DNA under the guidance of sequence-specific crRNAs, this process is called cis-cleavage. Additionally, Cas12a could form a Cas12a/crRNA/target DNA ternary complex, which activates the collateral cleavage activity of Cas12a, resulting in indiscriminate cleavage of nearby single-stranded DNA molecule, this is called trans-cleavage (Garcia-Doval and Jinek, 2017; Chen et al., 2018). Doudna et al. innovatively combines the cleavage activity exhibited by Cas12a with recombinase polymerase amplification (RPA, an isothermal amplification technology, which can exponentially amplify the target DNA at 37°C-42°C without thermal cyclers) technology. They developed a detection platform called DETECTR (DNA Endonuclease-Targeted CRISPR Trans Reporter), which achieves attomolar sensitivity of DNA detection. DETECTR can successfully distinguish 2 subtypes of human papillomavirus, HPV16 and HPV18, within 1 h (Chen et al., 2018). Wang et al. developed a HOLMES (one-Hour Low-cost Multipurpose highly Efficient System) detection platform by integrating PCR and Cas12a cleavage activity (Li et al., 2018). The establishment of these CRISPR-based detection platforms has greatly promoted the rapid development of next-generation molecular diagnostic technologies (Li et al., 2019). Currently, Cas12a-based detection technology has been successfully applied to the detection of various pathogens, including SARS-CoV-2 (Broughton et al., 2020; Guo et al., 2020; Talwar et al., 2021), norovirus (Qian et al., 2021), Salmonella and its drug resistance genes (Fu et al., 2022) etc.

**Figure 1:**
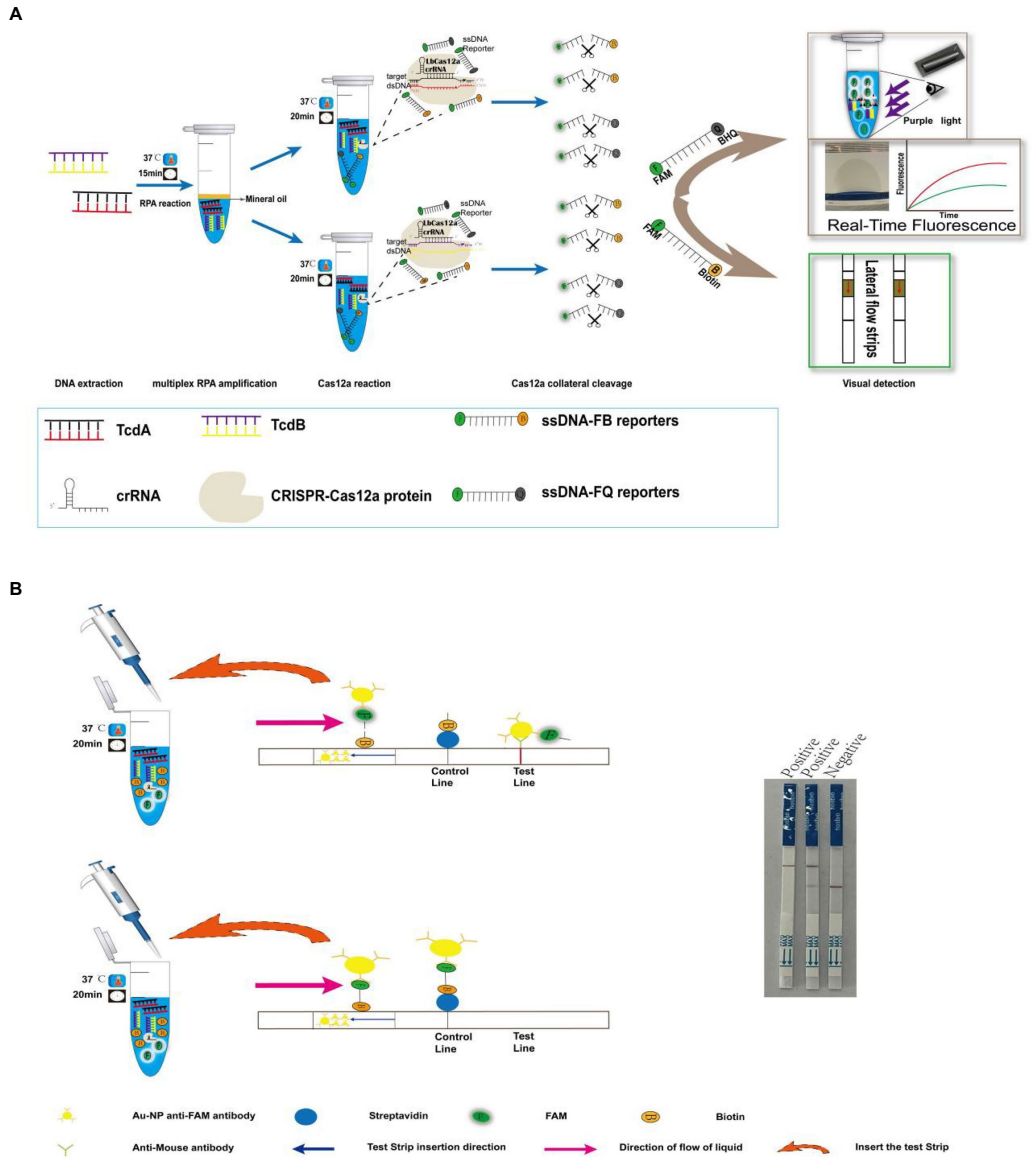
Multiplex RPA combined with Cas12a technology for *C. difficile* toxin gene detection strategy. **(A)** Schematic overview of the rapid detection of *C. difficile* dual toxin genes by multiplex RPA combined with Cas12a technology. **(B)** Schematic diagram of the principle of multiplex RPA-Cas12a-LFS.

In this study, we upgraded RPA isothermal amplification to multiplex RPA isothermal amplification, and developed a detection platform for *C. difficile* dual toxin gene by combining with CRISPR-Cas12a technology (Figure 1). To achieve visual detection, our method includes multiplex RPA-Cas12a-fluorescence detection and multiplex RPA-Cas12a-LFS detection. For the fluorescence detection, both real-time fluorescence and end-point fluorescence were included. This study is the first report investigating multiplex RPA combined with Cas12a technology for the detection of *C. difficile* toxin genes. We believe this platform enables rapid, highly sensitive, and cost-effective detection of *C. difficile* toxin genes, with potential application in point-of-care testing (POCT).

## Materials and methods

### Reagents and instruments

All primers, dual-labeled single-stranded DNA reporters (ssDNA-FQ, ssDNA-FB) were synthesized by Sangon Biotech Co., Ltd. (Shanghai, China). The crRNAs were synthesized by Shanghai GeneBio Co., Ltd. (Shanghai, China). The sequences of all the synthesized oligonucleotides are listed in Supplementary Table S1. Cas12a protein was purchased from Tolo Biotechnology Co., Ltd. (Shanghai, China). The TwistAmp Basic Kit for multiplex RPA was purchased from TwistDx (Cambridge, United Kingdom). Cas12a test strips were purchased from Warbio Biotechnology Co., Ltd. (Nanjing, China). The PUC57 plasmid was purchased from Sangon Biotech Co., Ltd. Bacterial DNA extraction kit and PCR product purification kit were purchased from Sparkjade Biotech (Shandong, China). Fecal genomic DNA extraction kit was purchased from Tiangen Bioch Co., Ltd. (Beijing, China). *C. difficile* toxin A/B gene detection kit was purchased from Hongweitest Biotechnology Co., Ltd. (Jiangsu, China). *C. difficile* chromogenic plates were purchased from Comagal Microbial Biotechnology Co., Ltd. (Shanghai, China). GENbag atmosphere generators were purchased from bioMérieux Inc. (Shanghai, China). Nucleic acid cleaners were purchased from GeneralBio Biotechnology Co., Ltd. (Anhui, China). The instrument used for isothermal amplification in this study was a mini dry bath, the fluorescence curve was monitored on a quantitative PCR (ABI7500), and the electrophoresis results were observed by a gel imaging system (BioRad, United States). Other small devices applied in this study include metal baths, microcentrifuges, and gun heads. All of these devices are suitable for *in situ* testing.

### Strains and DNA extraction

A total of 15 strains were investigated in this study were listed in Table 1. The positive control strain used in this study were clinical isolates, whose DNA was extracted after anaerobic culture. Our PCR results confirmed the positive control strain was a dual-toxin-producing strain (Supplementary Figure S1). *Escherichia coli O157: H7* (ATCC 700728), *Shigella flexneri* (CMCC (B) 51572), *Shigella dysentery* (CMCC (B) 51105), *Shigella sonnei* (CMCC (B) 51592), *Yersinia enterocolitica* (CMCC (B) 50024), and *Plesiomonas shigelloides* (ATCC 14029) were purchased from Shifeng Biotechnology Co., Ltd. (Shanghai, China). The rest of the strains were clinical isolates preserved in our laboratory (Identification by mass spectrometry or serological typing).

**Table 1:** Bacterial strains involved in this study.

Bacteria	Source	Fluorescent detection	LFS detection
*C. difficile*	Clinical isolates	P	P
*Escherichia coli O157:H7*	ATCC 700728	N	N
*Shigella flexneri*	CMCC (B) 51572	N	N
*Shigella dysentery*	CMCC (B) 51105	N	N
*Shigella sonnei*	CMCC (B) 51592	N	N
*Salmonella paratyphi beta*	Clinical isolates	N	N
*Salmonella paratyphi C*	Clinical isolates	N	N
*Salmonella typhi*	Clinical isolates	N	N
*Salmonella typhimurium*	Clinical isolates	N	N
*Yersinia enterocolitica*	CMCC (B) 50024	N	N
*Vibrio parahaemolyticus*	Clinical isolates	N	N
*Aeromonas hydrophila*	Clinical isolates	N	N
*Listeria monocytogenes*	Clinical isolates	N	N
*Plesiomonas shigelloides*	ATCC 14029	N	N
*Bacillus cereus*	Clinical isolates	N	N

CMCC, China Medical Culture Collection; ATCC, American Type Culture Collection; P, positive; N, negative.

All the bacterial strains were cultured before single colony was inoculated into a turbidity of 2.0 McFarland, then the DNA was extracted according to the instruction of the bacterial genomic DNA extraction kit.

### Design of primers and crRNAs

RPA primers were designed using Primer Premier 6 Primer Design Software (version 6.0; PREMIER Biosoft, United States). The sequences of tcdA and tcdB genes were acquired from NCBI, and aligned using SnapGene software (version 6.0.2; Dotmatics, United States) to identify the conserved sequence between tcdA and tcdB genes. According to their respective conserved sequences, 6 pairs of RPA primers for tcdA gene and 4 pairs of tcdB gene were designed (Supplementary Table S1), and the specificity of the primers was evaluated using NCBI-BLAST online tool, BLAST results showed that all of them had good specificity. Screening of RPA primers was performed using the TwistAmp^®^ Basic Kit (TwistDx).

To design the crRNA, we first searched for the protospacer adjacent motif PAM sequence (5′-TTTN) recognized by Cas12a in the conserved sequences of tcdA and tcdB genes. In order to improve the cleavage efficiency, we designed the crRNAs between the locations of forward and reverse primers, or overlapped with the primer sequences (Supplementary Table S1). The binding positions of primers and crRNA are shown in Supplementary Figure S2A. The structures of the two crRNAs are shown in Supplementary Figures S2B,C, which contain a conserved stem-loop structure and a spacer complementary to the target DNA sequence. The crRNAs bind to Cas12a *via* the stem-loop structure.

### Single RPA

RPA was performed using the TwistAmp^®^ Basic Kit (TwistDx) according to the manufacturer’s instructions with slight modifications. Briefly, the reaction solution includes rehydration buffer, forward and reverse primers, DNA template, and nuclease-free water to a volume of 47.5 μL. The above ingredients were added into a TwistAmp^®^ tube containing lyophilized enzyme. Finally, 2.5 μL of 280 mM magnesium acetate was added onto the inner side of the tube lid, the reaction was activated after brief centrifugation and subsequent incubation at 37°C for 20 min. The amplified product was purified with a PCR product purification kit and analyzed by 2% agarose gel electrophoresis. To avoid aerosol contamination due to opening the lid, 50 μL of paraffin oil is added to the surface of the reaction mixture to seal the aerosol.

### Multiplex RPA

Multiplex RPA were established based on single RPA. The multiplex RPA reaction was performed using the TwistAmp^®^ Basic Kit (TwistDx) with 50 μL reaction system, including rehydration buffer, 5 μM forward and reverse primers for tcdA, 5 μM forward and reverse primers for tcdB, DNA template, and nuclease-free water. The above ingredients were added into a TwistAmp^®^ tube containing lyophilized enzyme. Finally, 2.5 μL of 280 mM magnesium acetate was added onto the inner side of the tube lid, the reaction was activated after brief centrifugation and subsequent incubation at 37°C for 20 min. The amplified product was purified with a PCR product purification kit and analyzed by 2% agarose gel electrophoresis.

### Multiplex RPA-Cas12a-fluorescence assay

After the template DNA was amplified by multiplex RPA, the products were transferred to two Cas12a systems containing toxin A gene and B gene-specific crRNA, respectively. The tubes were immediately placed in the ABI7500 real-time PCR system for 20 min at 37°C where the fluorescence kinetics were recorded. The fluorescence intensity was recorded and curved every minute. End-point fluorescence was excited by a portable violet flashlight, and the images were captured by a smartphone.

After optimization, the 20 μL Cas reaction system of tcdA gene includes 500 nM Cas12a protein, 400 nM crRNA, 10 × TOLO buffer, 5 μM ssDNA-FQ, multiplex RPA product, and nuclease-free water. The 20 μL Cas reaction system of tcdB gene includes 300 nM Cas12a protein, 100 nM crRNA, 10 × TOLO buffer, 5 μM ssDNA-FQ, multiplex RPA product, and nuclease-free water.

### Multiplex RPA-Cas12a-LFS assay

Lateral flow strip (LFS) has received increasing attention in POCT due to its rapidness, convenience, and low equipment requirement (Zheng et al., 2021). Here, we used the optimized Cas system to replace the ssDNA-FQ reporter with the ssDNA-FB reporter, which remained a concentration of 5 μM. The 5′ and 3′ ends of the ssDNA were modified with FAM and Biotin, respectively. When the LFS-Cas12a reagents of the tcdA gene and tcdB were combined with the multiplex RPA products, they were incubated in a mini dry bath at 37°C for 20 min. After the incubation, 30 μL of nuclease-free water was added to the reaction tube to a final volume to 50 μL, before inserting a LFS (Tiosbio, Nanjing, China). After 3–5 min, visible band could be observed at the quality control area and the detection area of the strip.

### Sensitivity assessment

The conserved sequence of tcdA and tcdB genes was inserted into the *C. difficile* dual toxin gene plasmid, and the initial copy number of the plasmid was calculated (which was 10^10^ copies). Plasmid DNA was serially diluted 10 times, and multiplex RPA-Cas12a-fluorescence assay and multiplex RPA-Cas12a-LFS assay were performed to detect plasmid DNA of different copy numbers, which further determined the minimum detection limit of tcdA gene and tcdB gene.

### Specificity assessment

The specificity of the multiplex RPA-Cas12a-fluorescence assay and multiplex RPA-Cas12a-LFS assay was validated with 14 diarrhea-causing enteric pathogens, from which total DNA was extracted and used as templates.

### Clinical sample validation

A total of 10 diarrhea samples were collected from inpatients in different departments in the North District of the First Affiliated Hospital of Anhui Medical University from December 2021 to June 2022. Due to the anaerobic nature of the bacteria, all samples are stored at −80°C. All the samples were screened by anaerobic culture method and identified by mass spectrometer as *C. difficile*, after the samples were thawed, DNA was extracted using a fecal genome extraction kit according to the instruction manual. *C. difficile* toxin A/B quantitative PCR method was used as the reference method according to the manufacturer’s instructions. A dual toxin gene positive was confirmed by the FAM channel (tcdA gene) and the CY5 channel (tcdB) gene showing an obvious S-shaped amplification curve while the Ct value was less than 38. The clinical application potential of our established CRISPR/Cas12a-based detection method was verified by comparison with the reference method.

## Results

### Multiplex RPA-Cas12a-fluorescence/LFS assay strategy

As shown in Figure 1A, genomic DNA was extracted from clinical stool samples, after RPA amplification, the products were transferred to the Cas12a detection system containing tcdA gene and tcdB gene-specific crRNA, respectively. With the guidance of specific crRNA, the Cas12a protein recognized the PAM sequence and bond to *C. difficile* DNA, which activates the trans-cleavage activity of Cas12a. The activated Cas12a indiscriminately cleaved ssDNA which contains fluorescein and quencher molecules. For *C. difficile*-positive samples containing tcdA gene or tcdB gene, fluorescence will appear after Cas cleavage. Meanwhile, the FQ double-labeled ssDNA could be replaced to FB double-labeled ssDNA, which could be detected using LFS.

On the LFS, for negative samples, the ssDNA-FB probe remains intact, and the FAM labeled at one end binds to the anti-FAM antibody conjugated to gold nanoparticles (AuNPs). With the micro-flow, the reagent reaches the control area, the biotin labeling at the other end of the probe is captured by streptavidin, which accumulates the AuNPs that showing chromatographic change. For the positive samples, the free probes flow to the test area, where the anti-FAM antibody binds to the goat anti-mouse antibody, which accumulates the AuNPs that showing chromatographic change in the test area (Figure 1B).

### Primer optimization for multiplex RPA

**Figure 2:**
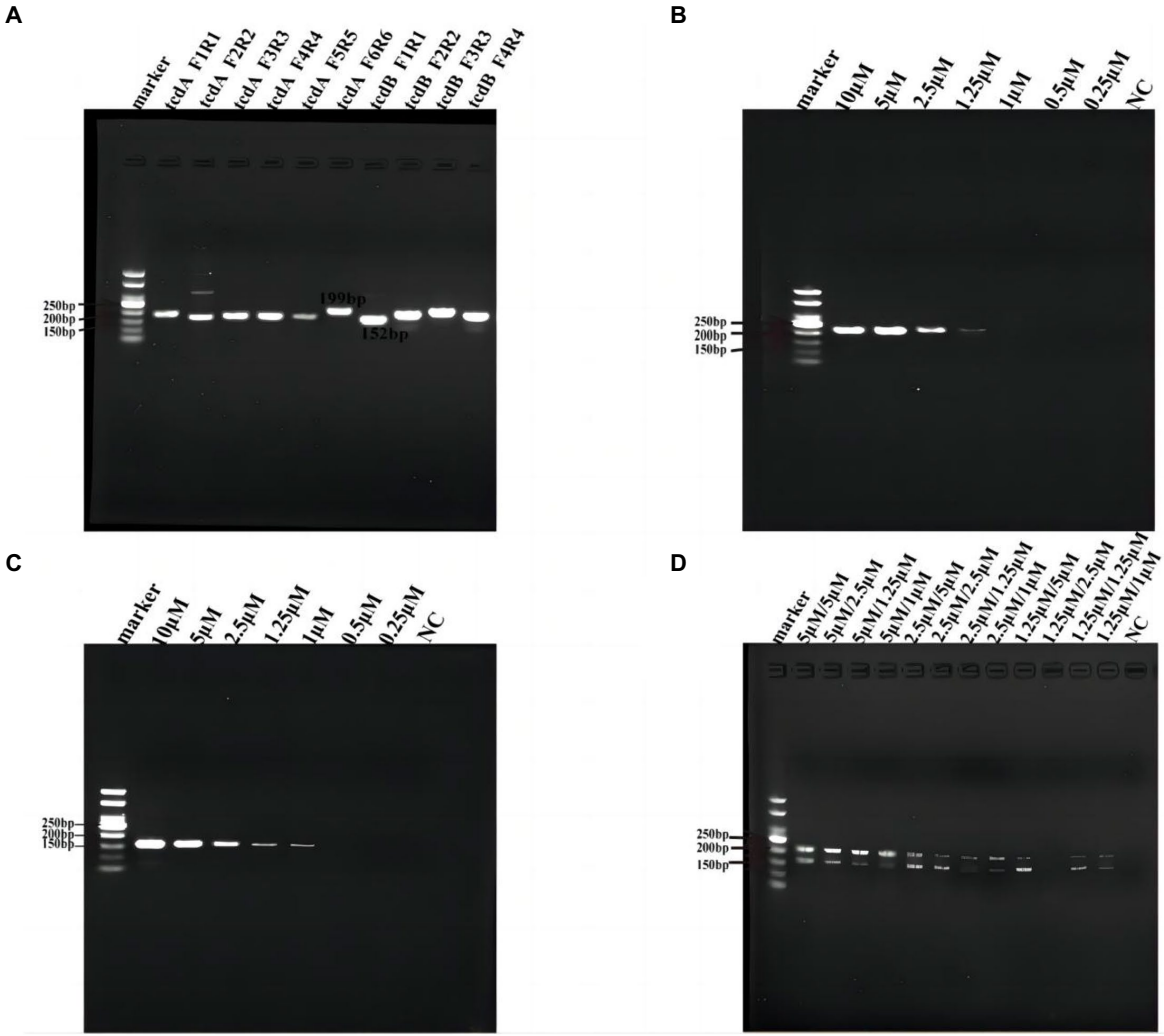
Primer optimization for multiplex RPA. **(A)** Six pairs of primers for tcdA and 4 pairs of primers for tcdB were tested by RPA. **(B)** Optimization of primer concentration for tcdA gene. **(C)** Optimization of primer concentration for tcdB gene. **(D)** Primer concentration cross-screening. NC, negative control, which nuclease-free water as template for RPA.

All the designed primers successfully amplified the template DNA. The optimal RPA primers were selected according to the highest intensity of the bands, where tcdA-F6/R6 and tcdB-F1/R1 were used as the primers for the following multiplex RPA (Figure 2A).

Then we optimized the primer concentrations for multiplex RPA since the amplification efficiency of different genes may vary if multiple pairs of primers coexisted in the same reaction tube, resulting in biased amplification and failure of multiplex RPA (Wang et al., 2021). We used the same concentration of forward and reverse primers for the two genes in RPA, and tested tcdA-F6/R6 and tcdB-F1/R1 at 0.25, 0.5, 1, 1.25, 2.5, 5, and 10 μM, respectively (Figures 2A,C). We found that for tcdA-F6/R6, the band was not clear when the concentration was lower than 1 μM (Figure 2B). For tcdB-F1/R1, no band was observed when the concentration was lower than 0.5 μM (Figure 2C). Next, we performed a concentration cross-screening experiment (Figure 2D) at 5, 2.5, and 1.25 μM for tcdA-F6/R6, and at 5, 2.5, 1.25, and 1 μM for tcdB-F1/R1. Our result showed that the intensity of DNA was the highest when both the concentrations of tcdA-F6/R6 and tcdB-F1/R1 were 5 μM. Therefore, primers at a concentration of 5 μM was used for multiplex RPA.

### Optimisation of the conditions of the Cas12a cutting system

**Figure 3:**
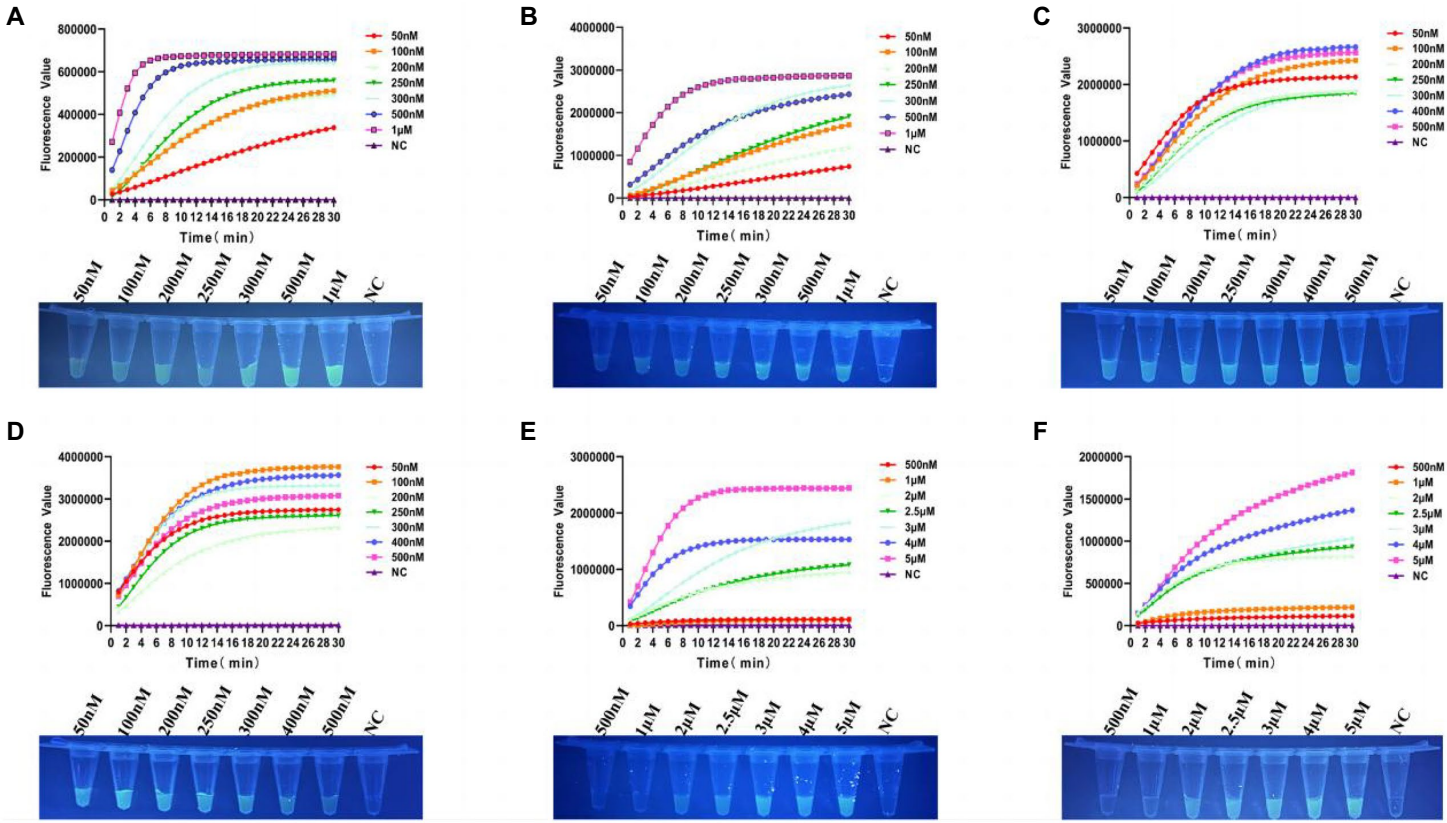
Optimization of the Cas12a reaction system. **(A)** Optimization of Cas12a concentration for tcdA gene. **(B)** Optimization of Cas12a concentration for tcdB gene. **(C)** Optimization of crRNA concentration for tcdA gene. **(D)** Optimization of crRNA concentration for tcdB gene. **(E)** Optimization of ssDNA-FQ concentration for tcdA gene. **(F)** Optimization of ssDNA-FQ concentration for tcdB gene. NC, negative control. All experiments were repeated at least 3 times.

In order to achieve the best detection performance, we optimized the Cas12a reaction system by fluorescence kinetics. First, we optimized the concentrations of Cas12a and crRNA (Figures 3A,B). With a fixed concentration of crRNA at 50 nM, we tested a series of Cas12a concentrations at 50, 100, 200, 250, 300, 500 nM, and 1 μM. From the amplification curve and the end-point fluorescence value of both tcdA and tcdB, we found that the optimal Cas12a concentration was 1 μM. Consistently, the fluorescence intensity illuminated by the UV flashlight was corresponding to the results from qPCR. Similarly, with a fixed concentration of Cas12a at 1 μM, we tested crRNA at 50, 100, 200, 250, 300, 400, and 500 nM. The amplification curve and the end-point fluorescence value showed that the optimal crRNA concentrations for tcdA and tcdB were 400 and 100 nM, respectively (Figures 3C,D). Subsequently, the ssDNA-FQ reporter concentration was optimized by comparing ssDNA-FQ at 500 nM, 1, 2, 2.5, 3, 4, and 5 μM. We found that the optimal ssDNA-FQ concentration for both tcdA and tcdB was 5 μM (Figures 3E,F).

### Sensitivity of multiplex RPA-Cas12a-fluorescence assay

**Figure 4:**
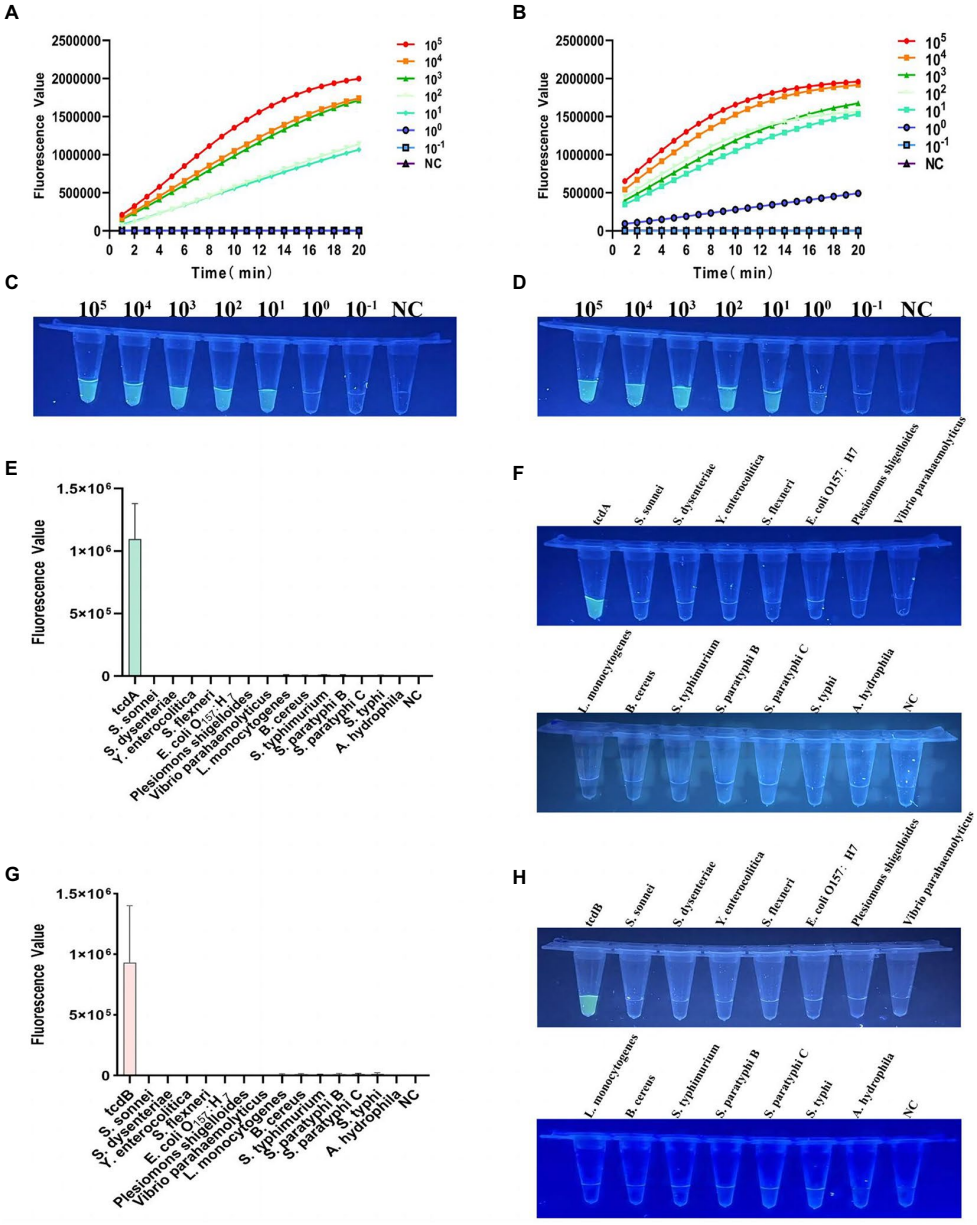
Verification of sensitivity and specificity of the multiplex RPA-Cas12a-fluorometric assay. **(A)** Sensitivity of tcdA gene after multiplex RPA amplification of the plasmid DNA with gradient dilution, fluorescence graphs. **(B)** Sensitivity of tcdB gene after multiplex RPA amplification of the plasmid DNA with gradient dilution, fluorescence graphs. **(C)** Sensitivity of tcdA gene after multiplex RPA amplification of the plasmid DNA with gradient dilution, endpoint Blueprint. **(D)** Sensitivity of tcdB gene after multiplex RPA amplification of the plasmid DNA with gradient dilution, endpoint blueprint. **(E)** Specificity of multiplex RPA amplification of tcdA gene. **(F)** The end-point fluorescence of tcdA gene after irradiation with a UV flashlight. **(G)** Specificity of multiplex RPA amplification of tcdB gene. **(H)** The end-point fluorescence of tcdB gene after irradiation with a UV flashlight. NC, negative control. All experiments were repeated at least 3 times.

As shown in Figure 4A, for the detection of tcdA, the multiplex RPA-Cas12a-fluorescence assay could detect a minimum of 10 copies of *C. difficile* plasmid DNA within 20 min. The end-point fluorescence showed consistent result that the minimum detectable number of tcdA as 10 copies (Figure 4C). For the detection of tcdB, plasmid DNA as low as 1 copy could be detected within 20 min (Figure 4B). The end-point fluorescence showed that weak fluorescence was visible with 1 copy, which was significantly different from the negative control (Figure 4D). Based on the visible end-point fluorescence detection by UV irradiation, the fluorescence was distinguishable with 1 copy of the template DNA, which validates the high sensitivity of our multiplex RPA-Cas12a-fluorescence assay.

### Specificity of multiplex RPA-Cas12a-fluorescence assay

For specificity analysis, we used multiplex RPA-Cas12a-fluorescence assay to detect the extracted genomic DNA of 14 clinical common diarrhea pathogens. As shown in Figures 4E,G, only tcdA and tcdB showed high fluorescent signals, which were visible to the naked eye, while other pathogens showed very low fluorescent values, indicating our method had no cross-reactivity with other pathogens. The endpoint fluorescence also specifically distinguished tcdA and tcdB (Figures 4F,H).

### Specificity and sensitivity of the multiplex RPA-Cas12a-LFS assays

**Figure 5:**
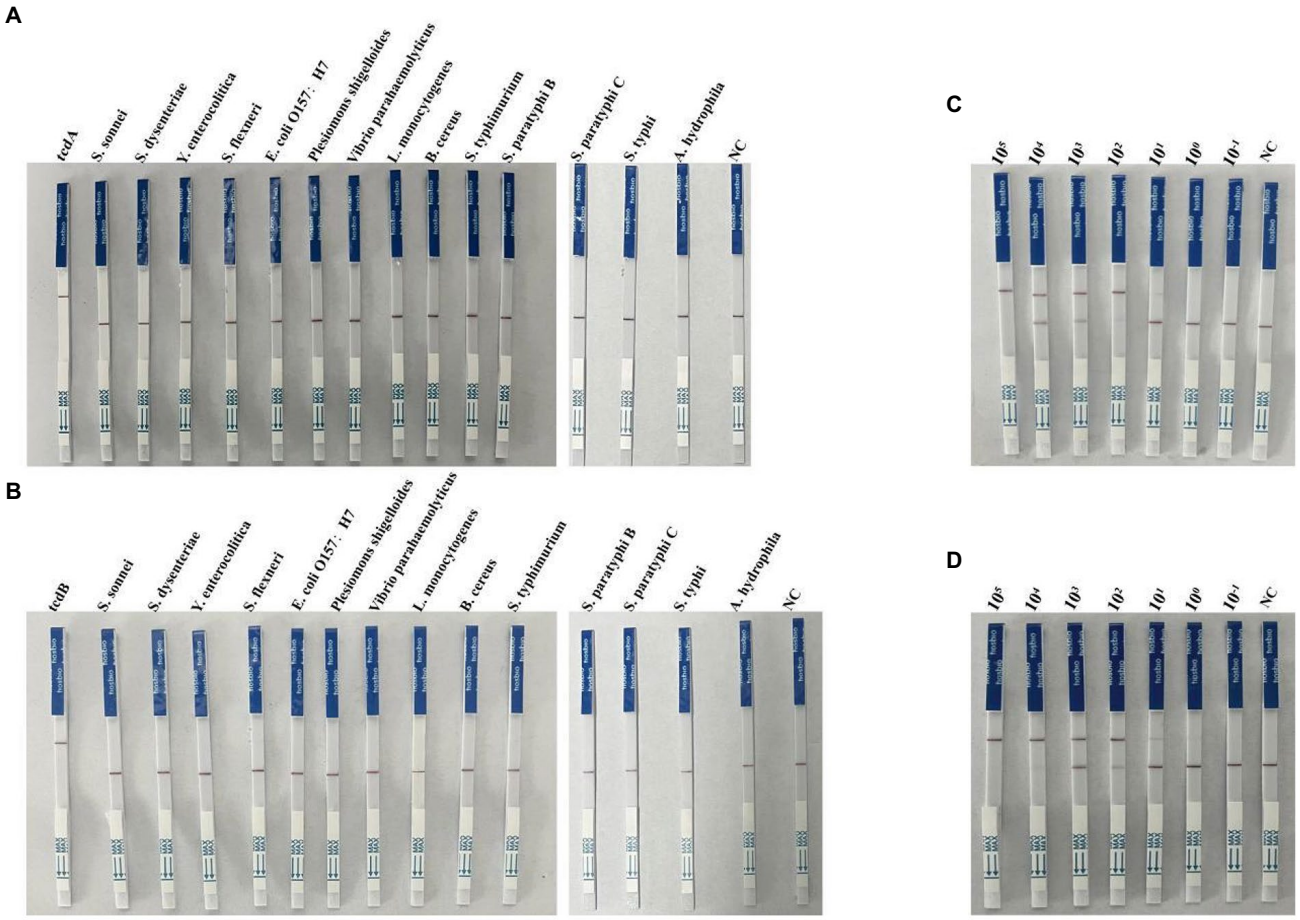
Determination of specificity and sensitivity of the multiplex RPA-Cas12a-LFS. **(A,B)** Specificity analysis of multiplex RPA-Cas12a-LFS. **(C,D)** Sensitivity analysis of multiplex RPA-Cas12a-LFS for tcdA gene **(C)** and tcdB gene **(D)**. NC, negative control. All experiments were repeated at least 3 times.

To evaluate the specificity of LFS assay, we detected the DNA samples extracted from 14 pathogenic bacteria after RPA amplification and Cas cleavage. Our results showed that only the test lines for tcdA and tcdB showed chromatographic change, the other assays were all negative (Figures 5A,B). For sensitivity analysis, we found a faint band in the test line with when 10 copies of tcdA (Figure 5C). The test line showed a faint band visible to the naked eye with 1 copy of tcdB (Figure 5D). Taken together, the sensitivity and specificity of the multiplex RPA-Cas12a-LFS assay were consistent with the fluorescent assay.

### Optimization of overall detection time

Since detection time is critical for POCT, we optimized the detection time of the multiplex RPA. We used 10^5^ and 10^2^ copies of plasmid DNA as template for the optimization. We first tested the multiplex RPA reaction time as 5, 10, 15, and 20 min. Our results showed that with 10^2^ copies of plasmid DNA, the fluorescence for tcdA gene was not detectable after 10 min (Supplementary Figure S3A), while the fluorescence of the tcdB gene appeared after 5 min of amplification (Supplementary Figure S3B). For the 10^5^ copies of plasmid DNA, both tcdA and tcdB showed strong fluorescence after multiplex RPA amplification for 5, 10, 15, and 20 min, respectively (Supplementary Figures S3C,D). Considering the low copy number in clinical samples, the multiplex RPA reaction time was set as 15 min. Next, we optimized the Cas12a cleavage time (Supplementary Figure S4). After 10 min of Cas12a cleavage, the tubes showed strong fluorescent signal (Supplementary Figures S4C,D). However, when we verified this condition using clinical samples, the fluorescent curve the curve increased significantly after 20 min (Supplementary Figure S6A) due to the low DNA concentration. Therefore, we kept the time of the Cas cleavage at 20 min. Similarly, we also optimized reaction time for Cas12a-LFS assay (Supplementary Figure S5). Our results showed that the optimal detection condition was multiplex RPA reaction for 15 min, Cas12a fluorescence analysis for 20 min, and Cas12a-LFS analysis for 20 min.

### Detection of *Clostridioides difficile* double toxin gene by Cas12a in clinical samples

**Figure 6:**
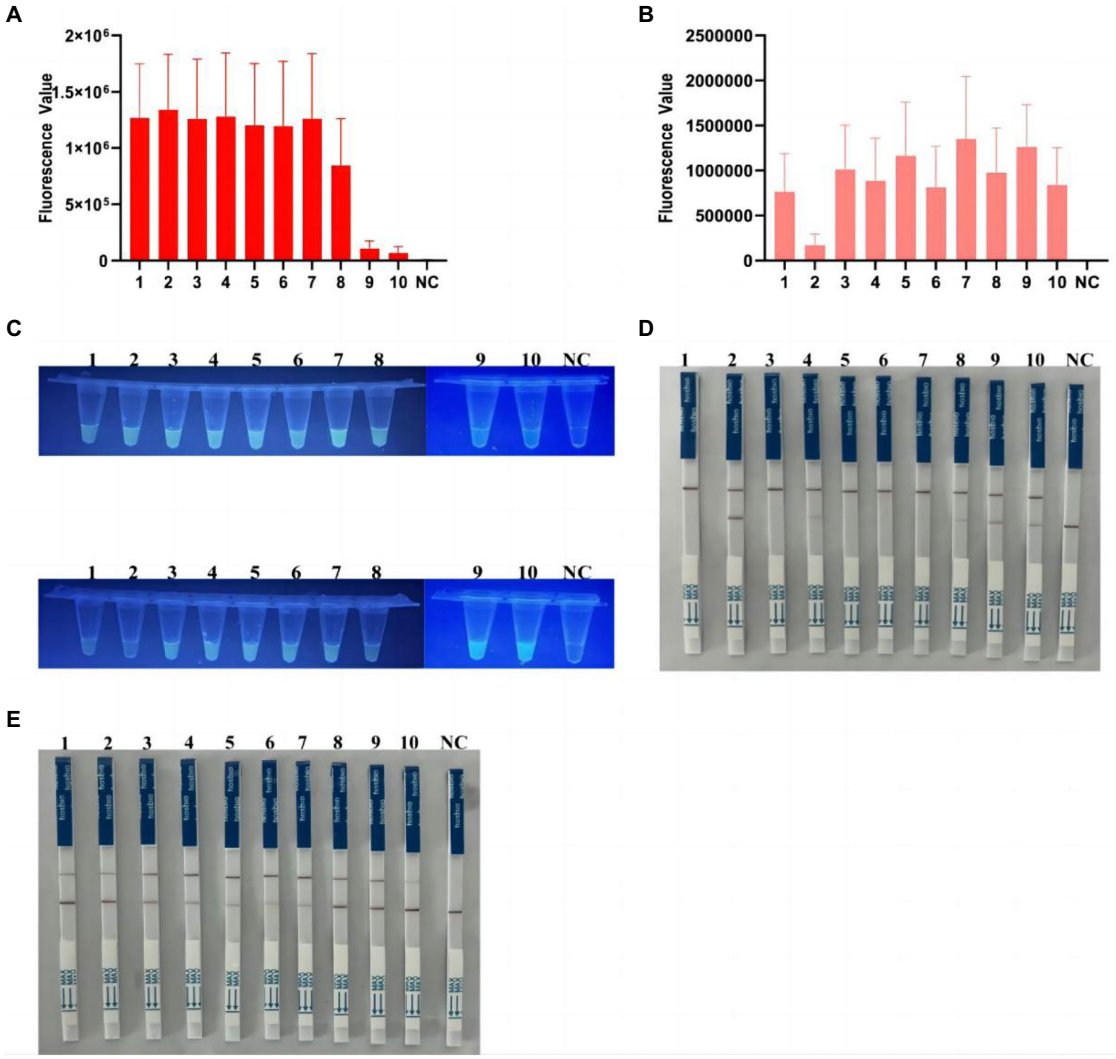
Detection of *C. difficile* dual toxin gene in clinical samples based on CRISPR-Cas12a. **(A)** Ten clinical samples were amplified by multiplex RPA for Cas12a fluorescence analysis of tcdA gene. **(B)** Ten clinical samples were amplified by multiplex RPA for Cas12a fluorescence analysis of tcdB gene. **(C)** End-point fluorescence for tcdA and tcdB in 10 clinical samples. All samples showed visible fluorescence. **(D,E)** Multiplex RPA-Cas12a-LFS assay was used to detect tcdA and tcdB genes in 10 clinical samples. All sample test lines were visible. NC, negative control. All experiments were repeated at least 3 times.

After multiplex RPA amplification and Cas12a cleavage in these samples, the dual toxin gene could be detected (Figures 6A,B and Supplementary Figures S6A,B). Visible fluorescence could be observed after irradiation by UV flashlight (Figure 6C). The test strips also clearly showed that 10 samples were double toxin gene-positive (Figures 6C,E), indicating our method could accurately identify *C. difficile* toxins A and B. In addition, we compared the Cas12a-based method with the commonly used quantitative PCR method for the detection of *C. difficile* toxin A and B. The quantitative PCR results showed that all 10 clinical samples contained the two toxin genes (Supplementary Figures S6C,D), showing S-shaped amplification curve, and the Ct values were all ≤35 (Supplementary Table S2). Therefore, our results were 100% consistent with the quantitative PCR results.

In addition, to further demonstrate the clinical applicability of our platform, we collected an additional 8 positive and 20 negative samples (excluding recent antibiotic use, etc.), which showed that the platform has good clinical applicability, with no fluorescence or negative LFS in the negative samples (Supplementary Figures S7–S9 and Supplementary Table S3).

## Discussion

*Clostridioides difficile* is an anaerobic, spore-forming, pathogenic toxin-producing bacterium that causes a wide variety of intestinal diseases, ranging from mild diarrhea to life-threatening pseudomembranous colitis. *C. difficile* has become the most common cause of hospital diarrhea (Kociolek and Gerding, 2016). With the emergence of the hypervirulentstrain BI/NAP1/027, *C. difficile* infection has become a deadly threat to global public health. At present, the diagnosis of *C. difficile* in most laboratories mainly relies on anaerobic culture or the detection of toxin A/B. Recently, a variety of rapid ELISA detection kits for toxin detection are commercially available, but both the sensitivity and specificity of these kits need to be improved. Anaerobic cultivation takes too long and cannot distinguish toxigenic and non-toxigenic strains. Although PCR-based toxin diagnostic methods can achieve high sensitivity and specificity, there is currently no standardized toxin A/B PCR detection kit approved for clinical application. Besides, the PCR-based methods require expensive equipment, which does not suitable for POCT.

In this study, we developed an efficient and portable *C. difficile* dual-toxin gene detection method with multiplex RPA combined with CRISPR/Cas12a, which can be used for the rapid diagnosis of *C. difficile* infection in patients. We used the CRISPR/Cas12a method to detect the toxin A gene (tcdA) and the toxin B gene (tcdB), and developed multiple detection platforms, including visible fluorescent detection using portable UV flashlight and LFS analysis combined with immunochromatography. These methods facilitate rapid on-site testing by reducing the equipment requirements, testing time, and cost. During the entire detection process, the isothermal amplification part is carried out in a small thermostatic metal bath apparatus. In addition, based on the UV flashlight irradiation, the fluorescence was clearly distinguishable with 1 copy of the plasmid DNA (Figure 4). Similarly, in the detection combined with LFS, the sensitivity could reach a minimum of 1 copy (Figure 5), indicating our method has a very high sensitivity, which is comparable to or even better than the PCR method. During the clinical sample validation, we compared with the quantitative PCR method, and the detection results of our method reached 100% consistency with the fluorescent PCR method (Figure 6 and Supplementary Figure S6). Taken together, our method is a potential, efficient method for the detection of the *C. difficile* gene.

Undeniably, there are some limitations in our method. Firstly, after performing the multiplex RPA amplification, the lid of the reaction tubes had to be opened for product transferring, which may increase the chance of aerosol contamination and the probability of false positive results. Secondly, although multiplex RPA method developed in this study could simultaneously detect two toxin gene, the subsequent Cas12a cleavage experiments still need to be operated separately, i.e., the multiplex Cas experiment is not feasible. On the other hand, only 10 clinical samples were used to verify the accuracy of the multiplex RPA method. Our ongoing study is to overcome these limitations, including developing non-opening operation and multiplex Cas detection platforms, and increasing the size of clinical samples. In conclusion, we developed a multiplex RPA combined with CRISPR/Cas12a method for the detection of *C. difficile* dual toxin genes, which can be further used for ultrasensitive detection of toxin genes for field detection.

## Conclusion

In conclusion, the CRISPR-based *C. difficle* dual toxin genes detection platform established by us has the advantages of high sensitivity and specificity, simple operation and low cost, and is very suitable for point-of-care detection.

## Data availability statement

The datasets presented in this study can be found in online repositories. The names of the repository/repositories and accession number(s) can be found in the article/Supplementary material.

## Ethics statement

The studies involving human participants were reviewed and approved by the Ethics Committee of the North District of the First Affiliated Hospital of Anhui Medical University and the collection of stool specimens complied with the ethical standards for intestinal microecology research (batch number: PJ-YX2021-021). Written informed consent to participate in this study was provided by the participants’ legal guardian/next of kin. The animal study was reviewed and approved by the Ethics Committee of the North District of the First Affiliated Hospital of Anhui Medical University and the collection of stool specimens complied with the ethical standards for intestinal microecology research (batch number: PJ-YX2021-021). Written informed consent was obtained from the owners for the participation of their animals in this study. Written informed consent was obtained from the individual(s), and minor(s)’ legal guardian/next of kin, for the publication of any potentially identifiable images or data included in this article.

## Author contributions

Material preparation, data collection, and analysis were performed by TJ, XH, CL, ZX, WY, YZ, HX, HT, and JS. The first draft of the manuscript was written by TJ wrote the first draft of the manuscript and all authors commented on previous versions of the manuscript. TJ, ZX, WY, YZ, HX, HT, and JS are participated in the study design and study management. TJ, ZX, XH, and CL participated in the collection of bacterial strains and the extraction of DNA. TJ and JS participated in data interpretation and writing of the manuscript. All authors contributed to the study conception and design, read and approved the final manuscript, have agreed on the publication of this manuscript, and agreed to be responsible for all their research work.

## Funding

This study was supported by 2020 Anhui Provincial University Cooperative Research and Public Health Collaborative Innovation Project of Anhui Provincial Department of Education (Grant No. GXXT-2020-016), 2021 Anhui Provincial Health and Health Commission Key Scientific Research Project (Grant No. AHWJ2021a011), 2021 Anhui Provincial Medical and Health Key Specialty Construction Project (Approval number: serial number 95), 2021 Anhui Provincial Key Project of Natural Science Research in Colleges and Universities (Approval number: KJ2021ZD0032).

## Conflict of interest

The authors declare that the research was conducted in the absence of any commercial or financial relationships that could be construed as a potential conflict of interest.

## Publisher’s note

All claims expressed in this article are solely those of the authors and do not necessarily represent those of their affiliated organizations, or those of the publisher, the editors and the reviewers. Any product that may be evaluated in this article, or claim that may be made by its manufacturer, is not guaranteed or endorsed by the publisher.
