# Supplementary material for: Rapid visualization of Clostridioides difficile toxins A and B by multiplex RPA combined with CRISPR-Cas12a
Source: Front Microbiol. 2023 Mar 8;14:1119395. doi: 10.3389/fmicb.2023.1119395 (PMC10030577; doi:10.3389/fmicb.2023.1119395)
Supplement: Supplementary file 1 [file Data_Sheet_1.docx]

Supplementary Material

**Rapid visualization of *Clostridioides difficile* toxins A and B by multiplex RPA combined with CRISPR-Cas12a**

Tong Jiang^1,2^,Xinyi Hu^1,2^,Chunhui Lin^1,2^, Zhaoxin Xia^1,2^, Wensu Yang^1,2^, Yi Zhu^1,2^, Huaming Xu^1,2^, Hao Tang^1,2^,and Jilu Shen^1^*

^1^The First Affiliated Hospital of Anhui Medical University, Hefei, Anhui, China, 230022

^2^Anhui Public Health Clinical Center, Hefei, Anhui, China, 230012

*** Correspondence:**

*Corresponding author:

Jilu Shen

Email: shenjilu@ahmu.edu.cn; 1464675852@qq.com

**Supplementary Table 1**. Sequence of primers/crRNAs/ssDNAs.

| **Primers/crRNA/ssDNA** | **Sequence (5'-3')** | **Product length** |
| --- | --- | --- |
| TcdA-RPA-F1 | TAATCATGGGATAGATATCAGGGCTAATAG | 172bp |
| TcdA-RPA-R1 | CTGGAAGCATATCAACATCTAAATATACTC |  |
| TcdA-RPA-F2 | CAGTACCTACAATAGATGATATTATAAAGTCTC | 153bp |
| TcdA-RPA-R2 | GTTCTGTAAACAAACTATTAGCCCTGATAT |  |
| TcdA-RPA-F3 | CTTTCGCTTTAGGCAGTGTTATAAATCAAG | 160bp |
| TcdA-RPA-R3 | TTAGTAGTATCTGTGAAGTTATTATCAGACTCT |  |
| TcdA-RPA-F4 | CAAATCAATAAACCTACAGTACCTACAATA | 164bp |
| TcdA-RPA-R4 | GTAAACAAACTATTAGCCCTGATATCTATC |  |
| TcdA-RPA-F5 | ATAGATATCAGGGCTAATAGTTTGTTTACA | 163bp |
| TcdA-RPA-R5 | CTGGAAGCATATCAACATCTAAATATACTC |  |
| TcdA-RPA-F6 | CATAGAGTCTGATAATAACTTCACAGATACT | 199bp |
| TcdA-RPA-R6 | ATTTATGAAATCATAGTAAGCTGACGCATA |  |
| TcdB-RPA-F1 | GAGAATCAATAAACTATACTGGTTGGTTAG | 152bp |
| TcdB-RPA-R1 | CTATTCACTAATCACTAATTGAGCTGTATC |  |
| TcdB-RPA-F2 | TCTTCTACTTCTCTGACTCTGGAATTATAG | 181bp |
| TcdB-RPA-R2 | TATTCAACTGCTTGTCCGTAAATATTATCA |  |
| TcdB-RPA-F3 | TCTTCTACTTCTCTGACTCTGGAATTATAG | 198bp |
| TcdB-RPA-R3 | CTCTAACTAAACCACTATATTCAACTGCTT |  |
| TcdB-RPA-F4 | TAATACACCAGATGGATTTAAATACTTTGC | 176bp |
| TcdB-RPA-R4 | AATAATACTCCTCACCATCAATAATAACTG |  |
| TcdA-crRNA | **UAAUUUCUACUAAGUGUAGAU** |  |
|  | AUGAUUCAUUAUUUAAUUCAG |  |
| TcdB-crRNA | **UAAUUUCUACUAAGUGUAGAU** |  |
|  | AUCCUGAUACAGCUCAAUUAG |  |
| ssDNA-FQ | FAM-TTATT-BHQ1 |  |
| ssDNA-FB | FAM-TTTTTTTATTTTTTT-Biotin |  |
| TcdA-PCR-F | CAGGGCTAATAGTTTGTTTACAGAACA[1] | 143bp |
| TcdA-PCR-R | CAACATCTAAATATACTCCGCCAAAA |  |
| TcdB-PCR-F | AGCAGTTGAATATAGTGGTTTAGTTAG [1] | 144bp |
| TcdB-PCR-R | CATGCTTTTTTAGTTTCTGGATTGAA |  |

TcdA, toxin A gene; TcdB, toxin B gene; bold font indicates the universal binding sequence of crRNA to Cas12a.


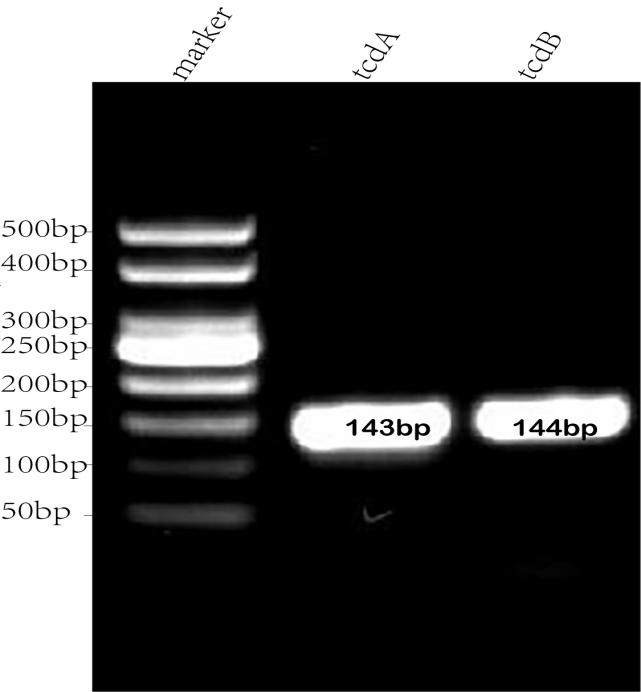


**Supplementary Figure S1** Validation of double-toxin production by PCR of positive strains.


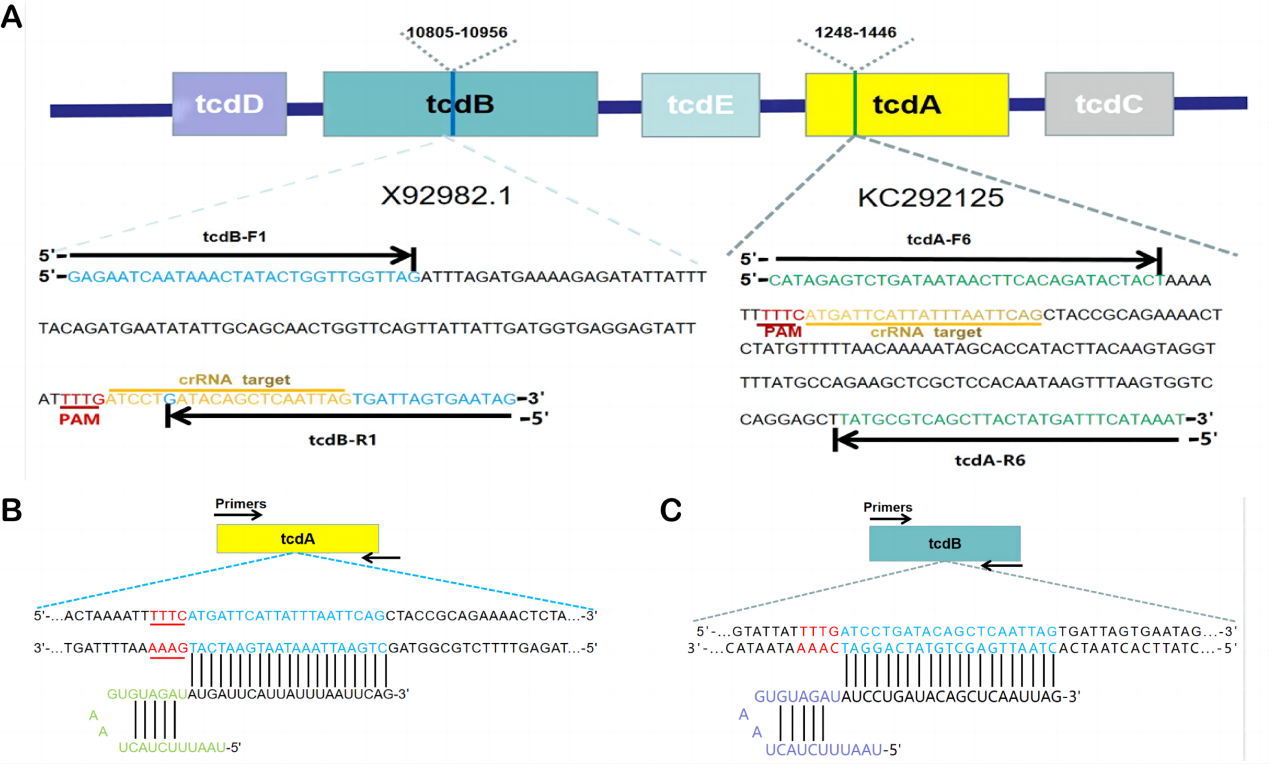


**Supplementary Figure S2**  Schematic diagram of the binding sites of multiplex RPA primers and crRNAs on the *C. difficile* toxin genes. (A) Binding sites of multiplex RPA primers in tcdA gene and tcdB gene. (B) TcdA-specific crRNA sequence and structure. (C) TcdB-specific crRNA sequence and structure.


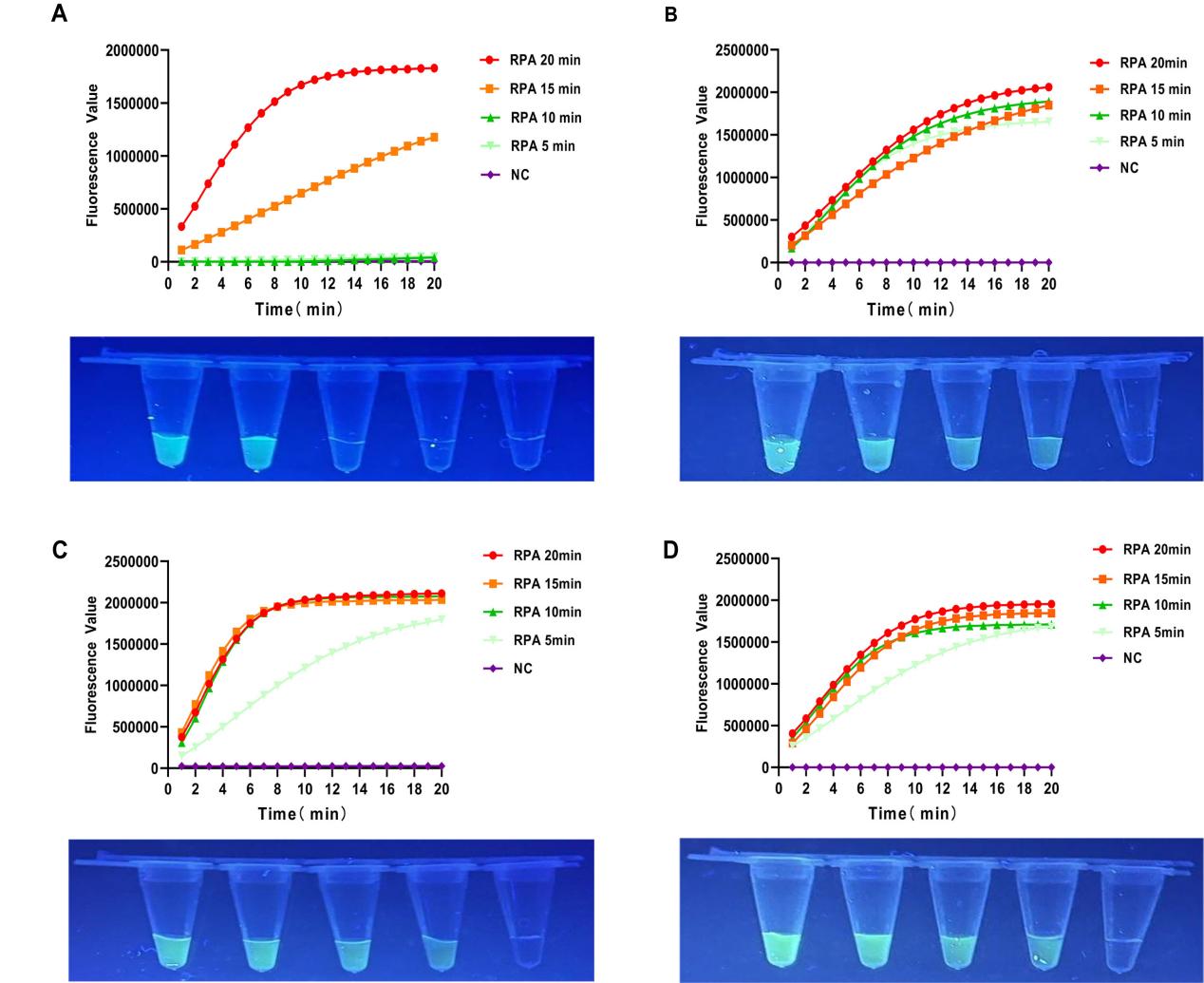


**Supplementary Figure S3** Optimization of multiplex RPA reaction time. (A) TcdA gene amplification curve and end-point fluorescence with multiplex RPA amplification for 10^2^ copies of plasmid DNA. (B) TcdB gene amplification curve and end-point fluorescence with multiplex RPA amplification for 10^2^ copies of plasmid DNA. (C) TcdA gene amplification curve and end-point fluorescence with multiplex RPA amplification for 10^5^ copies of plasmid DNA. (D) TcdB gene amplification curve and end-point fluorescence with multiplex RPA amplification for 10^5^ copies of plasmid DNA. NC, negative control.

**
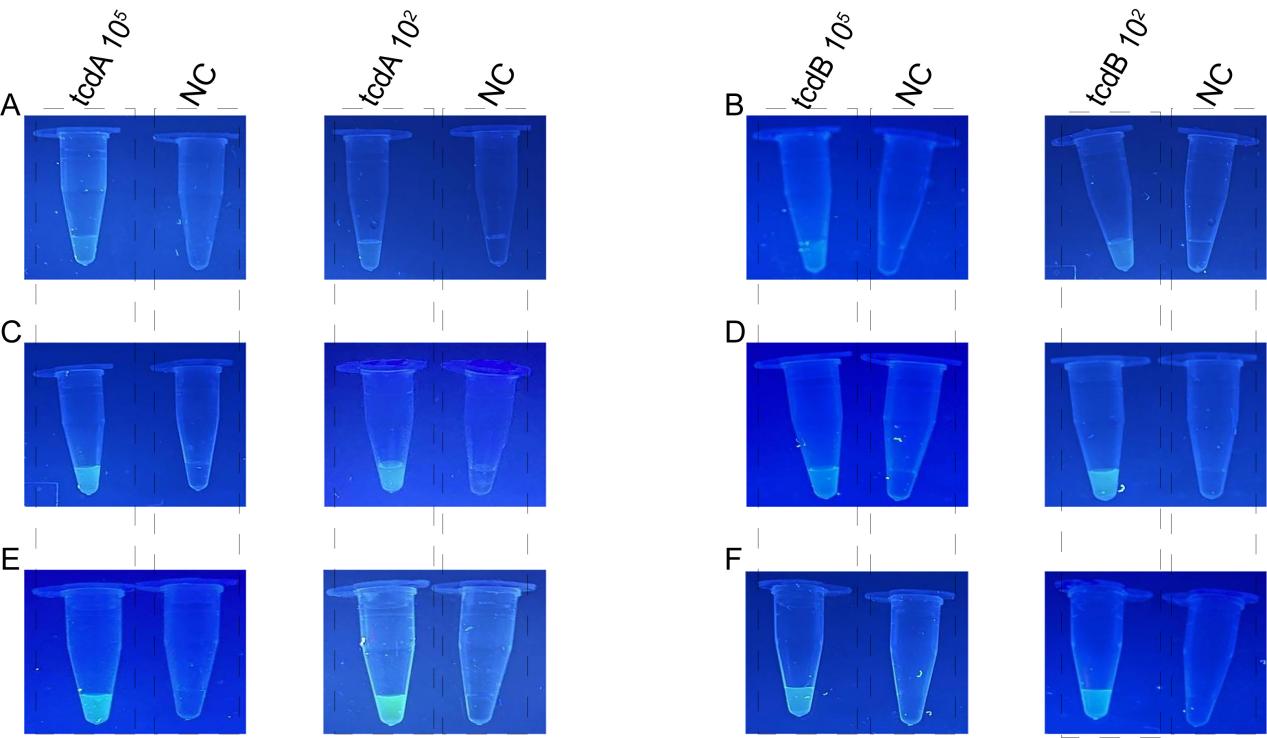
**

**Supplementary Figure S4** Optimization of Cas12a reaction time. (A) 10^5^ and 10^2^ copies of plasmid DNA were amplified by multiplex RPA for 15 min, respectively. Representative fluorescent images showing tcdA gene cleaved by Cas12a for 5 min. (B) 10^5^ and 10^2^ copies of plasmid DNA were amplified by multiplex RPA for 15 min, respectively. Representative fluorescent images showing tcdB gene cleaved by Cas12a for 5 min. (C) 10^5^ and 10^2^ copies of plasmid DNA were amplified by multiplex RPA for 15 min, respectively. Representative fluorescent images showing tcdA gene cleaved by Cas12a for 10 min. (D) 10^5^ and 10^2^ copies of plasmid DNA were amplified by multiplex RPA for 15 min, respectively. Representative fluorescent images showing tcdB gene cleaved by Cas12a for 10 min. (E) 10^5^ and 10^2^ copies of plasmid DNA were amplified by multiplex RPA for 15 min, respectively. Representative fluorescent images showing tcdA gene cleaved by Cas12a for 15 min. (F) 10^5^ and 10^2^ copies of plasmid DNA were amplified by multiplex RPA for 15 min, respectively. Representative fluorescent images showing tcdB gene cleaved by Cas12a for 15 min. NC, negative control.

**
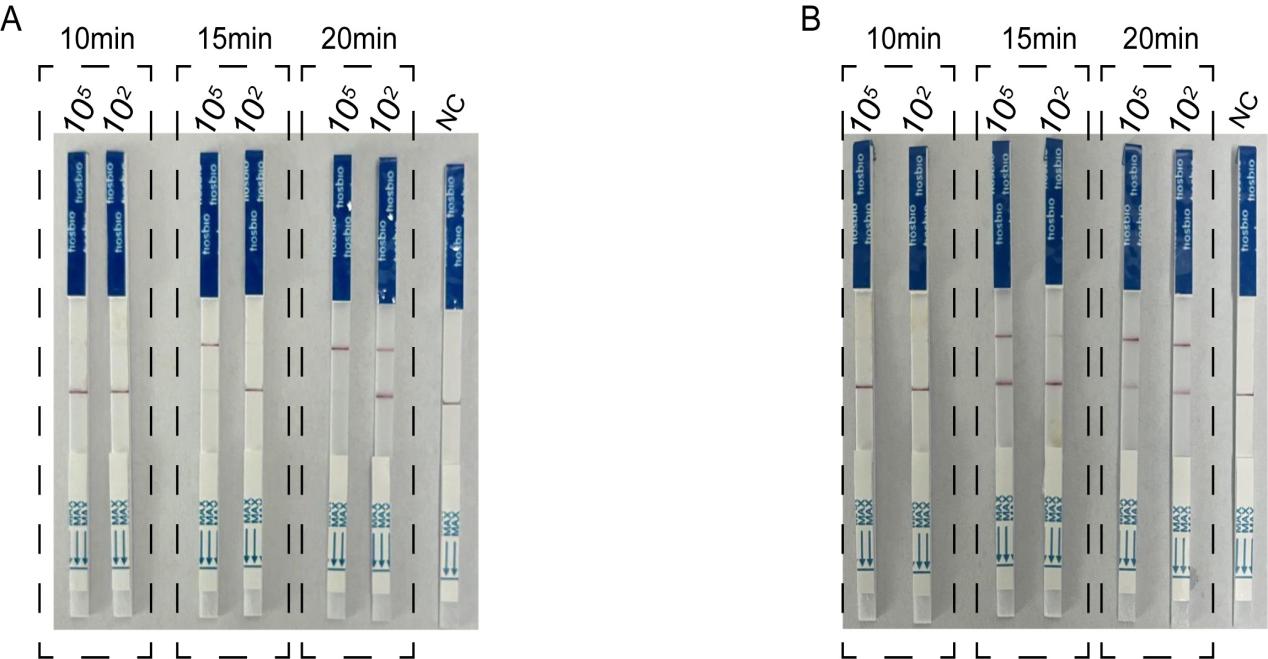
**

**Supplementary Figure S5** Optimization of Cas12a-LFS reaction time. (A) 10^5^ and 10^2^ copies of plasmid DNA were amplified by multiplex RPA for 15 min, respectively. Representative LFS images showing tcdA gene cleaved by Cas12a for 10 min, 15 min, and 20 min, respectively. (B) 10^5^ and 10^2^ copies of plasmid DNA were amplified by multiplex RPA for 15 min, respectively. Representative LFS images showing tcdB gene cleaved by Cas12a for 10 min, 15 min, and 20 min, respectively. NC, negative control.


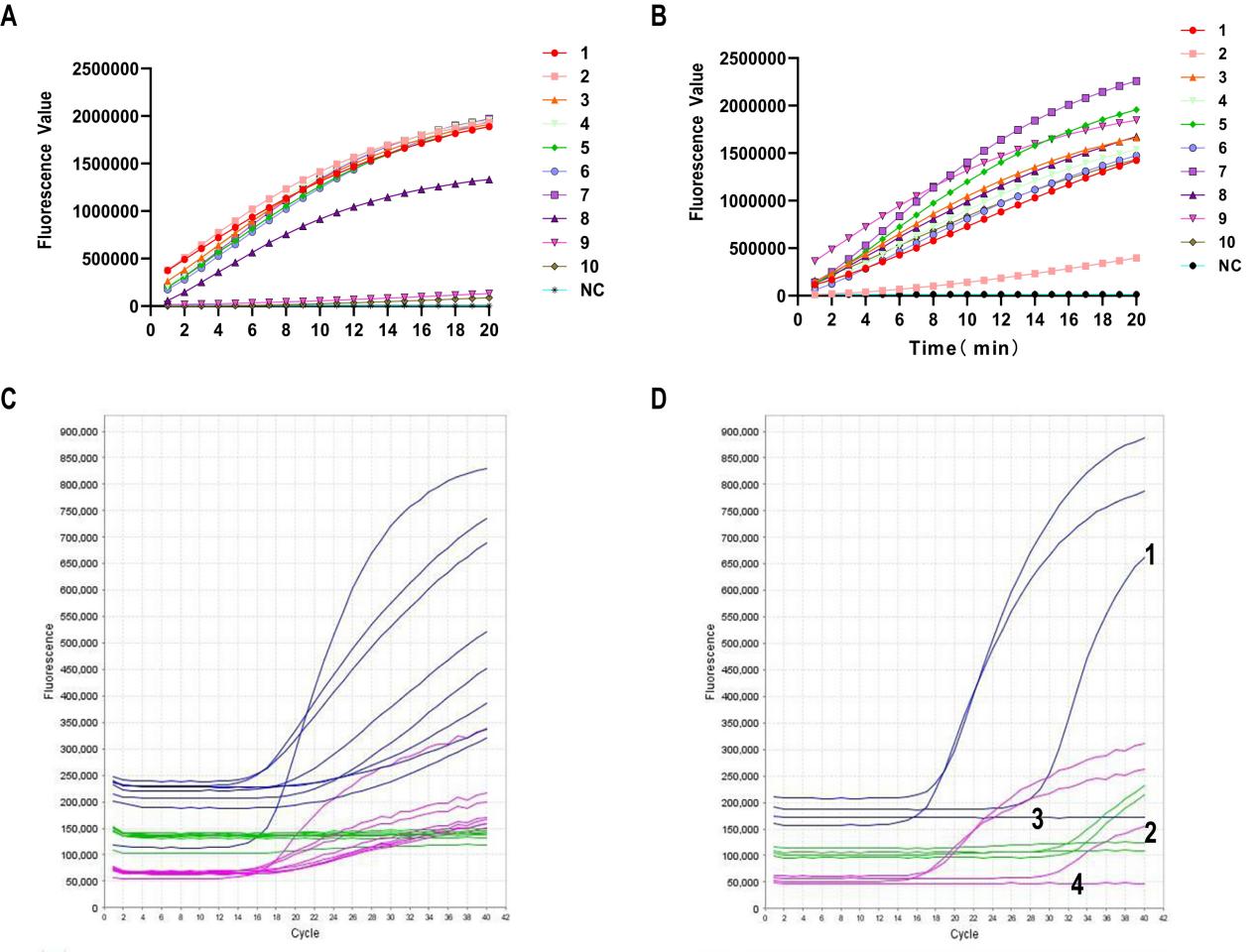


**Supplementary Figure S6** (A) Amplification curve of tcdA gene from 10 clinical samples with Cas cleavage. (B) Amplification curve of tcdB gene from 10 clinical samples with Cas cleavage. (C) and (D) represent the qPCR results of 10 clinical samples, in which the blue curves represent the amplification of tcdA gene, the purple curves represent the amplification of tcdB gene. The green curve represents the internal control. 1 represents the positive control for tcdA gene. 2 represents the positive control for tcdB gene. 3 represents the negative control for tcdA gene. 4 represents the negative control for tcdB gene.

**
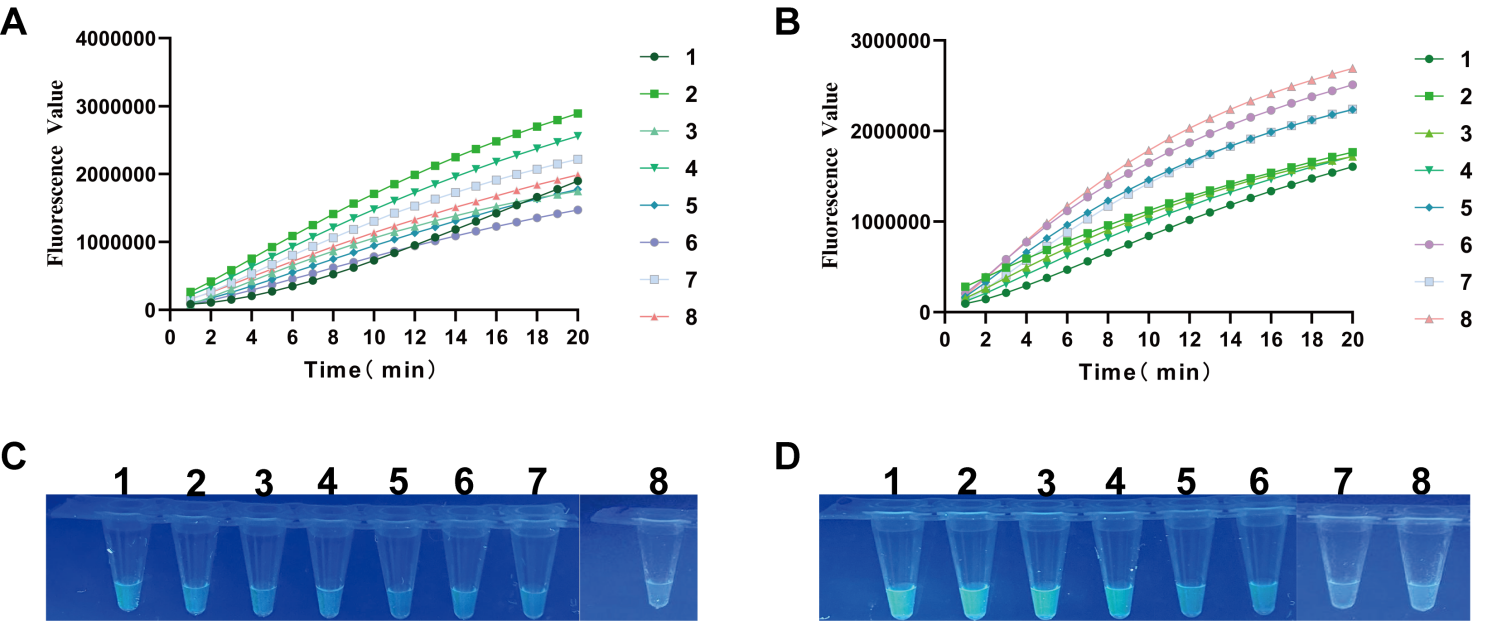
**

**Supplementary Figure S7** Fluorescence results from 8 positive samples. (A) Fluorescence curve of tcdA gene from 8 positive samples. (B) Fluorescence profile of tcdB gene for 8 positive samples. (C) Endpoint fluorescence map of the tcdA gene in eight positive samples under violet torch illumination. (D) Endpoint fluorescence map of 8 positive samples of tcdB gene under violet torch illumination.

**
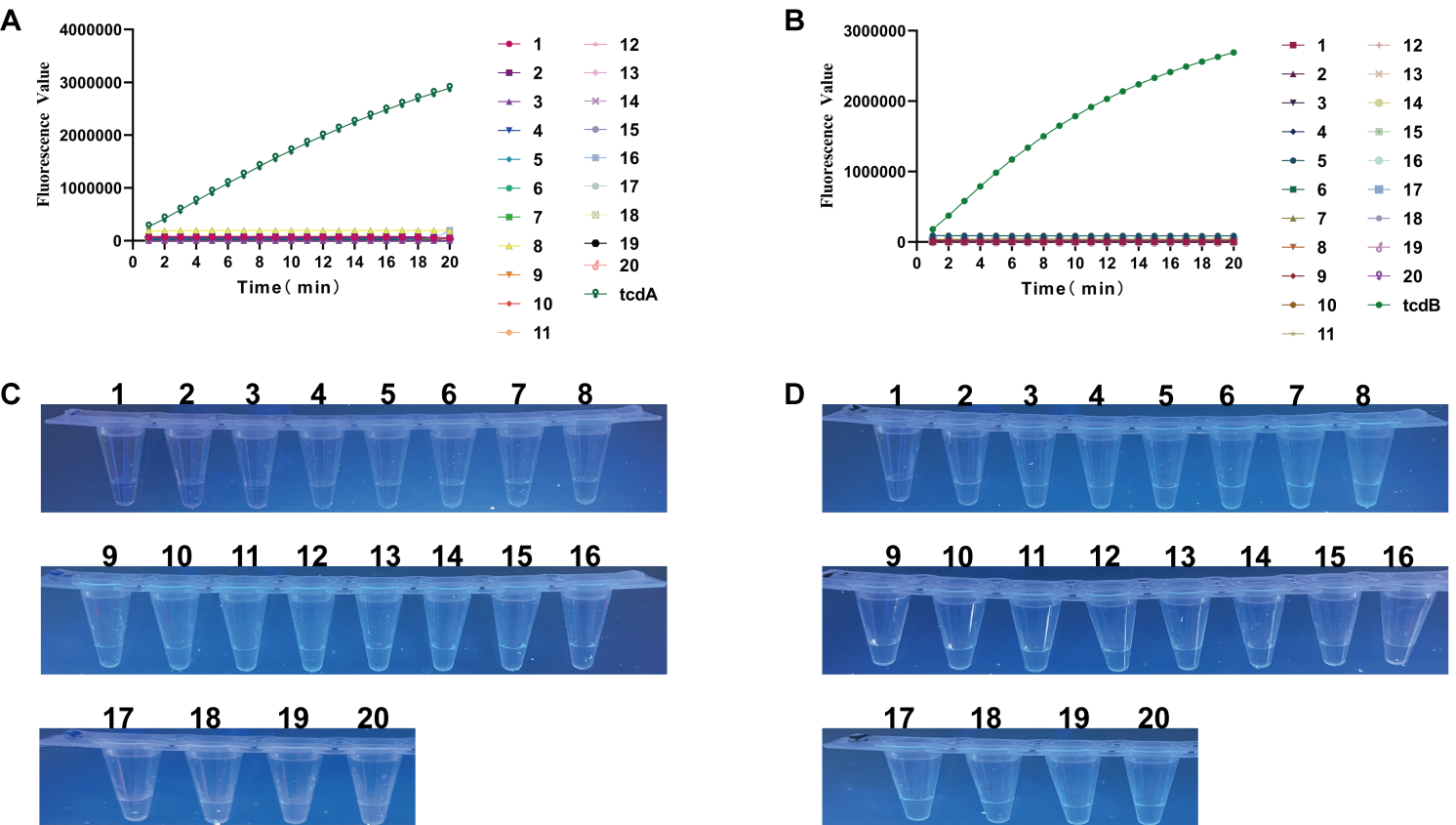
**

**Supplementary Figure S8** Fluorescence results for 20 negative samples. (A) Fluorescence profile of tcdA gene in 20 negative samples. (B) Fluorescence profiles of tcdB genes from 20 negative samples. (C) Fluorogram of tcdA gene endpoints from 20 negative samples. (D) Fluorogram of tcdB gene endpoints from 20 negative samples.

**
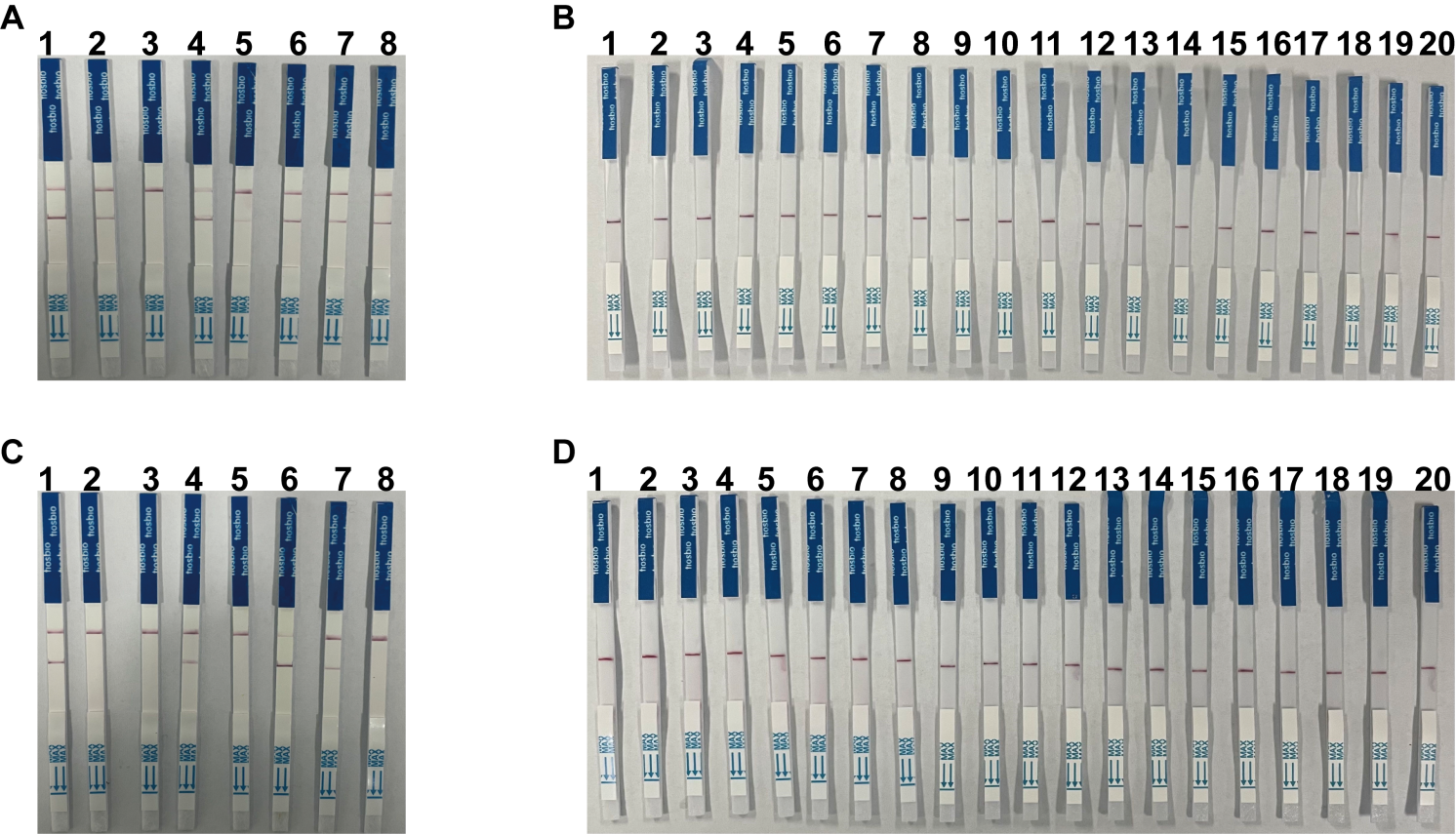
**

**Supplementary Figure S9** LFS results for 8 positive and 20 negative samples. (A) LFS results for tcdA gene in 8 positive samples. (B) LFS results for tcdA gene in 20 negative samples. (C) LFS results for tcdB gene in 8 positive samples. (D) LFS results for tcdB gene in 20 negative samples.

| **Sample** | **Ct value for tcdA** | **Ct value for tcdB** |
| --- | --- | --- |
| 1 | 15.03 | 15.51 |
| 2 | 23.03 | 19.54 |
| 3 | 18.48 | 17.94 |
| 4 | 21.13 | 18.99 |
| 5 | 21.05 | 20.04 |
| 6 | 22.91 | 20.55 |
| 7 | 15.53 | 15.94 |
| 8 | 15.23 | 15.45 |
| 9 | 16.86 | 17.06 |
| 10 | 15.47 | 15.55 |
| NC | / | / |

**Supplementary Table 2.** Ct values of 10 clinical samples by qPCR.

| **Sample** | **Ct value for tcdA** | **Ct value for tcdB** |
| --- | --- | --- |
| 1 | 15.83 | 16.54 |
| 2 | 12.54 | 13.0 |
| 3 | 13.97 | 13.42 |
| 4 | 12.58 | 14.46 |
| 5 | 15.46 | 14.95 |
| 6 | 11.38 | 12.98 |
| 7 | 16.04 | 13.55 |
| 8 | 31.12 | 33.85 |
| 9 | / | / |
| 10 | / | / |
| 11 | / | / |
| 12 | / | / |
| 13 | / | / |
| 14 | / | / |
| 15 | / | / |
| 16 | / | / |
| 17 | / | / |
| 18 | / | / |
| 19 | / | / |
| 20 | / | / |
| 21 | / | / |
| 22 | / | / |
| 23 | / | / |
| 24 | / | / |
| 25 | / | / |
| 26 | / | / |
| 27 | / | / |
| 28 | / | / |

**Supplementary Table 3.** Additional 8 positive samples and 20 negative samples were supplemented with qPCR results.

**Reference**

1. Wroblewski, D., et al., *Rapid molecular characterization of Clostridium difficile and assessment of populations of C. difficile in stool specimens.* J Clin Microbiol, 2009. **47**(7): p. 2142-8.
